# An efficient approach for feature construction of high-dimensional microarray data by random projections

**DOI:** 10.1371/journal.pone.0196385

**Published:** 2018-04-27

**Authors:** Hassan Tariq, Elf Eldridge, Ian Welch

**Affiliations:** 1 School of Engineering and Computer Science, Victoria University of Wellington, Wellington, New Zealand; 2 Department of Computer Science, University of Agriculture, Faisalabad, Pakistan; University of North Carolina at Chapel Hill, UNITED STATES

## Abstract

Dimensionality reduction of microarray data is a very challenging task due to high computational time and the large amount of memory required to train and test a model. Genetic programming (GP) is a stochastic approach to solving a problem. For high dimensional datasets, GP does not perform as well as other machine learning algorithms. To explore the inherent property of GP to generalize models from low dimensional data, we need to consider dimensionality reduction approaches. Random projections (RPs) have gained attention for reducing the dimensionality of data with reduced computational cost, compared to other dimensionality reduction approaches. We report that the features constructed from RPs perform extremely well when combined with a GP approach. We used eight datasets out of which seven have not been reported as being used in any machine learning research before. We have also compared our results by using the same full and constructed features for decision trees, random forest, naive Bayes, support vector machines and k-nearest neighbor methods.

## Introduction

Microarray is a collection of DNA or RNA attached to a solid surface. The purpose of the microarray is to do expression profiling or assessing the genome content in closely related cells or organisms [1]. Microarray datasets have become a center of attention for researchers working in bioinformatics and machine learning domains. Studying the underlying patterns of differential gene expression is a major challenge in these kinds of datasets, as the number of instances for both training and testing is usually less than 100, while on the other hand number of features ranges from 6000–60,000. High dimensionality implies high computational cost and massive memory requirements for training. The capacity of these trained algorithms is also compromised by what is known as the curse of dimensionality [2]. Several studies have been carried out to find a robust machine learning method to classify such data [3].

Evolutionary algorithms (EA) are population-based, random search techniques where a population of solutions gets updated iteratively using algorithm-specific heuristics until convergence is achieved [4]. Genetic programming(GP) is one of the most popular techniques among the EA community. Since GP's introduction by Koza [5], the research community has frequently applied it to solve problems such as optimization, control, data mining, image processing and signal processing [6]. Dimensionality reduction maps data to low-dimensional space from high-dimensional space by assuming that the intrinsic structure of the high-dimensional data can remain intact in the low-dimensional space. Principal Component Analysis (PCA) and Linear Discriminant Analysis (LDA) are the two most commonly used dimensionality reduction techniques. These two techniques construct features which perform well with various machine learning algorithms, but the high computational cost is one of the major limitations of these methods. To address this issue of computational cost, Random Projection (RP), which maps data to a randomly generated, low-dimensional latent space, was proposed [7]. The motivation behind the current work was to explore the effectiveness of RP for feature construction to improve the classification performance of a GP classifier for a high-dimensional microarray dataset. The purpose of this work was to address the following objectives;

1. To investigate the performance of GP on very high-dimensional microarray datasets.
2. To investigate the performance of random projection-based features constructed with GP.
3. To investigate how k-Nearest Neighbours(KNNs), Support Vector Machines(SVMs), Decision Trees(DT), Naive Bayes(NB) and Random Forests(RFs) algorithms perform on very high-dimensional microarray datasets as compared to GP.

## Background

GP is a population-based method to evolve programs [8]. It typically follows these steps:

1. *Initialization*: produce an initial population of programs from terminal and function sets.
2. Until a certain stopping-criteria is fulfilled, perform:
  - *Evaluation*: the fitness of each individual program is calculated by a pre-selected fitness function.
  - *Selection*: select a subset of programs to produce next generation of programs based on their fitness scores.
  - *Evolution*: generate new generation by either copying a program to the new generation (reproduction) or combining different parts of programs or mutating a part of a program randomly(crossover).
3. Return the solution with the highest fitness.

### Terminal and function set

In GP, each program is a tree-like structure where terminal nodes are the feature values and internal nodes are elements of a pre-determined function set, in our case (+, −, ÷, ×).

### Fitness function

In order to measure the fitness of our program, we used Mathew’s Correlation coefficient (MCC). The MCC is a correlation between the observation and prediction which in our case is defined as:

$$MCC=\frac{N_{tp}N_{tn}-N_{fp}N_{fn}}{\sqrt{\left(N_{tn}+N_{fn})(N_{tn}+N_{fp})(N_{tp}+N_{fn})(N_{tp}+N_{fp}\right)}}$$ (1)

Where N_tp_, N_tn_, N_fp_, and N_fn_ are the number of true positives (TPs), true negatives (TNs), false positives (FPs) and false negatives (FNs), respectively. When the denominator is 0, MCC is set to 0. The standardized fitness of a rule was calculated as:

$$fitness=\frac{1+MCC}{2}$$ (2)

Since MCC ranges between -1.0 and +1.0, the standardized fitness ranges between 0.0 and +1.0, the higher values being better with 1.0 being the best.

## Dataset description

For experimentation, we have chosen eight high-dimensional microarray datasets.

1. Lung Cancer Histology- This Dataset is a comparison of two non-small cell lung cancer histology sub-types [9]. It contains expression levels of 54,675 RNAs of 58 carcinoma's sample: 18 squamous cell carcinoma(SCC) and 40 adenocarcinomas(AC). The complete dataset is available at https://www.ncbi.nlm.nih.gov/sites/GDSbrowser? acc=GDS3627.
2. Oral Mucosa—This dataset provides insight into the carcinogenic effects of cigarette smoking [10]. The dataset has expression levels of 54675 RNAs of 79 Oral mucosa samples: 39 smokers and 40 non- smokers. The complete dataset is available at https://www.ncbi.nlm.nih.gov/sites/GDSbrowser?acc=GDS3709.
3. B lymphocytes-This dataset was generated during a study conducted on US white females [11]. The objective was to analyse the effect of smoking on circulating B lymphocytes because B cells are directly linked with the onset of smoking-induced diseases. The dataset contains expressions levels of 22,283 RNAs from 79 blood samples of females: 40 non-smokers and 39 smokers. The complete sets of data are available at https://www.ncbi.nlm.nih.gov/sites/GDSbrowser?acc=GDS3713.
4. Placenta dataset- This dataset provides an insight into the effect of tobacco smoking on placenta [12]. Smoking increases the risk of preterm delivery and other complications during pregnancy. The dataset has expression levels of 11,155 RNAs taken from the placenta of 76 females: 64 non-smokers and 12 smokers. The dataset is available at https://www.ncbi.nlm.nih.gov/sites/GDSbrowser?acc=GDS3793.
5. Melanoma- This dataset provides an insight into the molecular basis of primary melanoma and melanoma metastasis [13]. It has the microarray expression levels of 22,283 RNAs from 83 melanoma samples: 31 primary melanomas and 52 melanoma metastasis. The dataset is available at https://www.ncbi.nlm.nih.gov/sites/GDSbrowser?acc=GDS3966.
6. Breast cancer- It contains 97 cDNA microarrays, each representing 24,481 genes based on the biopsy specimens of primary breast tumors of patients with germline mutations of relapsed and non-relapsed. The pre-processed dataset is available at http://csse.szu.edu.cn/staff/zhuzx/Datasets.html.
7. Skeletal muscle-This dataset gives an overview of molecular changes in the skeletal muscles of young and old people [14]. The dataset has 54,675 RNA expression levels of 110 samples: (62) young and (48) old. The pre-processed dataset is available at https://www.ncbi.nlm.nih.gov/sites/GDSbrowser
8. Osteoarthritis- This dataset provides an insight into the molecular changes of Osteoarthritis patients [15]. It has 48, 802 RNA microarray expression from 139 patients: 106 osteoarthritis and 33 control. The pre-processed dataset is available at https://www.ncbi.nlm.nih.gov/sites/GDSbrowser

## Experimental set up

**Fig 1:**
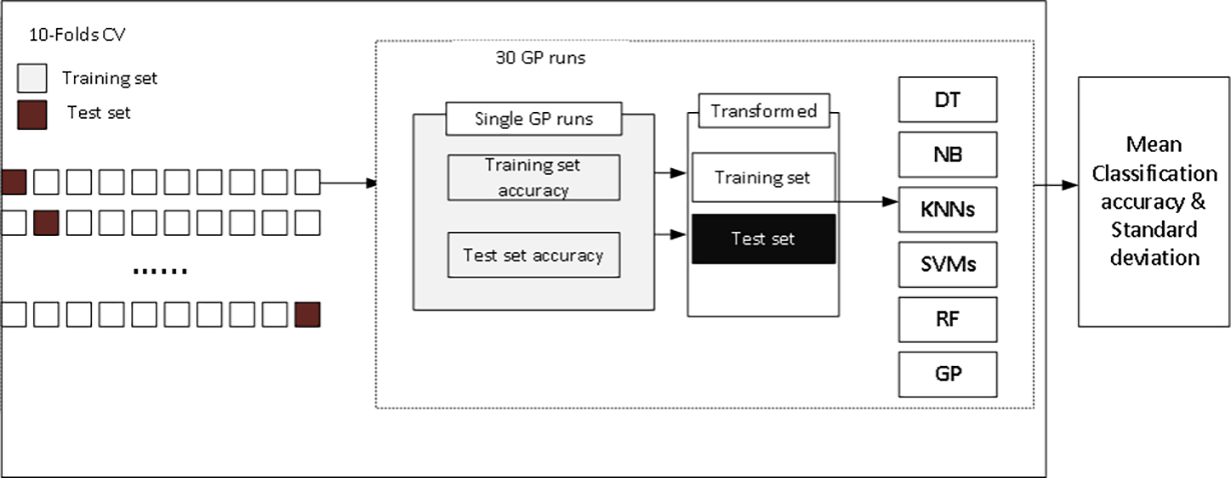
10-fold cross-validation for GP. Training set and Test set performance evaluations goes into Tables 3 and 4 respectively. Performance has been measured in each of the GP run for each fold and used to calculate mean accuracies and standard deviations by the end of 10-folds.

To measure the performance of our method for feature selection and classification, we conducted several experiments on the eight different microarray datasets. ECJ [16] was used for GP and Weka package [17] was used to implement random projections for feature construction. The Weka API was also used for KNNs, SVMs, DT, NB and RFs classifiers. We used K-fold cross-validation to avoid feature selection bias for all of the above methods, and the value of k is 10. Our experimental design is shown in Fig 1.

In random projection, if we have d-dimensional data originally then it is projected through the origin to a k-dimensional (k << d) subspace, using a random k*d matrix, R, whose columns have unit lengths [18]. Using matrix notation where Xd*N is the original set of N d-dimensional observations,

$$X_{k\times N}^{RP}=R_{k\times d}X_{d\times N}$$ (3)

Table 1, gives the summary of parameters used. Ramped half-and-half was used to generate the initial population of algorithms/RPs, where the individual tree depth ranges from 2 to 8. Tournament selection with size 7 and population of size 1024 was used. Elitism is applied to copy the best individual into next generation. Once the maximum number of generations is achieved, termination of the evolutionary process takes place. The whole experiment was repeated 30 times with random seeds.

**Table 1:** GP settings.

Function set	+, -, x, ÷
Terminal set	Feature values
Initialization method	Ramped half-and-half
Tree depth	2–8
Crossover probability	0.8
Mutation probability	0.2
Selection method	Tournament
Tournament size	7

We use accuracy to measure the performance of models on training and test sets. For training data, the performance is measured as:

TrainingSetAccuracy=MCC*100

And for test data the performance is measured as:

$$Test\;Set\;Accuracy=\frac{1}{2}\;\left(\frac{N_{tp}}{N_{tp}{+N}_{fn}}+\frac{N_{tn}}{N_{tn}{+N}_{fp}}\right)$$

## Results and discussion

We have used eight datasets, all of them have a very low number of instances and very large number of features. As we can see in Table 2**,** that shows the results of using GP with the full feature set, it has not given us good training accuracy as compared to other machine learning algorithms. In most of the cases, SVM and RF have achieved very good training accuracy results.

**Table 2:** Training set accuracies of GP and machine learning algorithms.

Dataset	Features	GP	DT	NB	KNNs	SVMs	RF
Adenocarcinomas (58)	54675	97.4 ± 2.2	99.04 ± 0.9	94.82±1.2	100± 0	100± 0	100± 0
Oral Mucosa(79)	54675	84.5±3.8	99.57 ± 0.9	98.45 ± 0.7	87.76± 1.3	100± 0	100± 0
B-Cells (79)	22283	89.8 ± 3.2	100± 0	99.71± 0.5	80.45±2.9	100± 0	100± 0
Placenta (76)	11155	83.3 ± 4.3	91.52 ± 4.7	80.55 ± 2.6	86.69±1.9	96.93±2.1	100± 0
Melanoma (83)	22283	97.3 ± 1.5	99.19 ± 0.65	100±0	92.5±1.6	100±0	100±0
Breast cancer (97)	24482	86±3	98.85 ±0.72	55.9± 3.6	75.25± 2.5	100± 0	100± 0
Skeletal Muscle (110)	54675	91 ± 4.4	99.39 ±0.8	99.09±0.5	96.36±0.6	100±0	100±0
Osteoarthritis (139)	48802	86.28±2.6	99.43 ± 0.6	71 ± 9.2	79.13± 1.2	100± 0	100± 0

Similar is the case when calculated the Test set accuracy as shown in Table 3**.**

**Table 3:** Test set accuracies of GP and machine learning algorithms.

Dataset	Features	GP	DT	NB	KNNs	SVMs	RF
Adenocarcinomas	54675	83 ± 15	83 ± 12.9	87.67 ± 11	89.67 ± 13.5	96.67± 6.67	89.67 ± 9.2
Oral Mucosa	54675	62±16	77.32 ± 12	74.82±13.5	72.14±9.3	82.5±15	76.78± 16
B-Cells	22283	69±16	80 ± 15	83.75 ± 13.7	72.5± 10.8	91.25±9.7	91.25±11.25
Placenta	11155	74 ± 11	71.24 ±15.6	73.92±14.5	78.92± 10.6	63.21±11.5	81.6±6.2
Melanoma	22283	86.8 ± 11	84.16 ± 11	92.91 ± 8	87.63±7.9	92.78 ±8	95.27 ± 5.8
Breast cancer	24482	57.2 ±16	63.11 ±18.8	54.66± 4.1	57.44 ± 19	68.11±14.9	71.11 ± 11
Skeletal Muscle	54675	79.39±13	86.36 ±10	92.72 ± 7.9	90 ±9.4	96.36 ± 7.2	96.36 ±8.1
Osteoarthritis	48802	73±11	86.2± 8.5	57.58 ±20	76.9 ± 4.2	92.08±5.9	78.4 ±5.5

SVMs has performed exceptionally well for almost all the high-dimensional datasets. For Skeletal Muscle and Adenocarcinoma datasets, it has achieved greater than 96%. RF has also achieved very good results with all the datasets. The most impressive of them are skeletal muscle and Melanoma datasets. In case of KNNs (k = 3), Skeletal Muscle, Adenocarcinoma, and Melanoma datasets have shown good results. For NB and DT, Melanoma and skeletal muscle have shown better results as compared to other datasets. With GP, Adenocarcinoma and Melanoma datasets have shown better performance from the rest of the datasets.

As shown in Table 4**,** the newly constructed features by using random projections have shown the different story as that of using full feature subset. We have constructed three sets of features for each of the datasets. GP has shown excellent results in all the cases. In case of 50 constructed features, GP has shown best results all the time. As the number of constructed features increase, the accuracy gradually decreases. But in case of other algorithms, the patterns are different. For Adenocarcinoma dataset, as we use a higher number of features average accuracy increases slowly. Most significant change is in case of DT and RF where there is a rise of 12% in average accuracy.

**Table 4:** Comparison of random projections based feature construction.

Dataset	Features	GP	DT	NB	KNNs	SVMs	RF
Adenocarcinomas(58)	50	99.83±0.009	73.33±1.6	87.66±3	86±1.3	91.33±0.7	77.67±1.5
	100	98.46±0.08	76.33±3.3	90±3.3	93.3±1.0	91.67±1.2	89.67 ± 1.5
	150	97.14±0.9	86±2.7	90±3.3	91.67±1.3	95±1.6	89.67±1.5
Oral Mucosa(79)	50	99.95±0.002	79.82±1.8	74.82±3.44	57.14±6.6	81±1.4	78.3±2.2
	100	98.57±0.08	70.89±0.16	69.46±3.8	62.14±2.1	77.32±2.6	81.25±5.9
	150	96.93±0.17	68.39±0.95	65.71±2.7	64.64±1.3	81.75±1.4	78.75±6.7
B-Cells(79)	50	99.41±0.03	77.32±2.6	74.82±3.44	82.32±2.2	85±4.7	82±5.5
	100	97.28±0.15	72.32±4.2	76.07 ±3.0	77.57±2.2	88.75±3.5	83.75±5.1
	150	96.59±0.19	71.25±9	78.57±2.2	76.25±6.7	87.5±3.9	83.75±5.1
Placenta(76)	50	99.91±0.005	77.49±2.5	68.75±9.8	70.71±4.7	84.28 ± 0.45	84.28±0.45
	100	99.30±0.03	73.39±5.1	67.5±10.7	69.46±5.1	84.28±0.45	84.28±0.45
	150	97.95±0.11	70.71±4.2	68.75±9.8	69.46±5.1	84.28±0.45	81.6±1.2
Melanoma(83)	50	97.64±0.13	88.19±4.1	94.16±1.8	95.13±2.4	91.67±1.3	96.52±1.0
	100	97.06±0.16	84.3±2.9	92.77±1.67	95.13±1.53	96.3±2.8	97.77±0.7
	150	96.37±0.51	85.5±3.3	95.2±1.4	96.3±1.14	97.63±0.7	97.77±0.7
Breast cancer(97)	50	97.87±0.12	52.77±0.8	49.44±1.9	43.22±0.3	54.66±0.2	56±0.14
	100	96.75±0.18	52.55±2.5	49.33±1.9	47.22±11	51.55±1.2	55.88±0.1
	150	96.90±0.17	51.67±2.2	51.44±1.3	52.1±2.4	52.55±0.9	57±0.45
Skeletal Muscle(110)	50	99.24±0.04	65.45±2.2	69.09±7.4	74.54±0.57	89.09±2.2	83.63±0.5
	100	98.69±0.07	71.81±5.4	66.36±2.0	83.63±5.1	98.18±0.57	82.72±0.28
	150	98.27 ±0.09	63.36±0.8	68.18±1.43	85.45±1.72	97.27±2.0	81.81±2.8
Osteoarthritis(139)	50	99.90±0.005	71.97±1.5	72.74±3.7	78.4±0.46	86.97±3.1	78.46±1.9
	100	99.23±0.04	70±9.4	69.12±2.4	84.23±2.5	90.65±0.52	81.31±1.04
	150	98.73±0.07	82.03±0.8	67.69±2.9	83.51±2.7	94.23±0.68	77.74±2.17

For Oral mucosa, 50 constructed features have shown better results for all the algorithms except KNNs and RF. when compared to 100 and 150 features. In case of B-Cells dataset, DT and KNNs have shown better results with a lower number of features along with GP. As for the highly unbalanced dataset of Placenta, the accuracy was maintained for all of the algorithms except DT. Most of the times, there is a very small difference in accuracy when using RF, NB, KNNs, and SVMs.

For Melanoma dataset and Breast cancer datasets, a higher number of constructed features show better results in all the methods except DT. In case of skeletal muscle dataset, KNNs and SVMs show better results as we increase the number of features while the inverse is true for DT, NB, and RF. In case of Osteoarthritis dataset, a higher number of features has shown better classification accuracies as compares to other feature subsets for all the methods.

**Fig 2:**
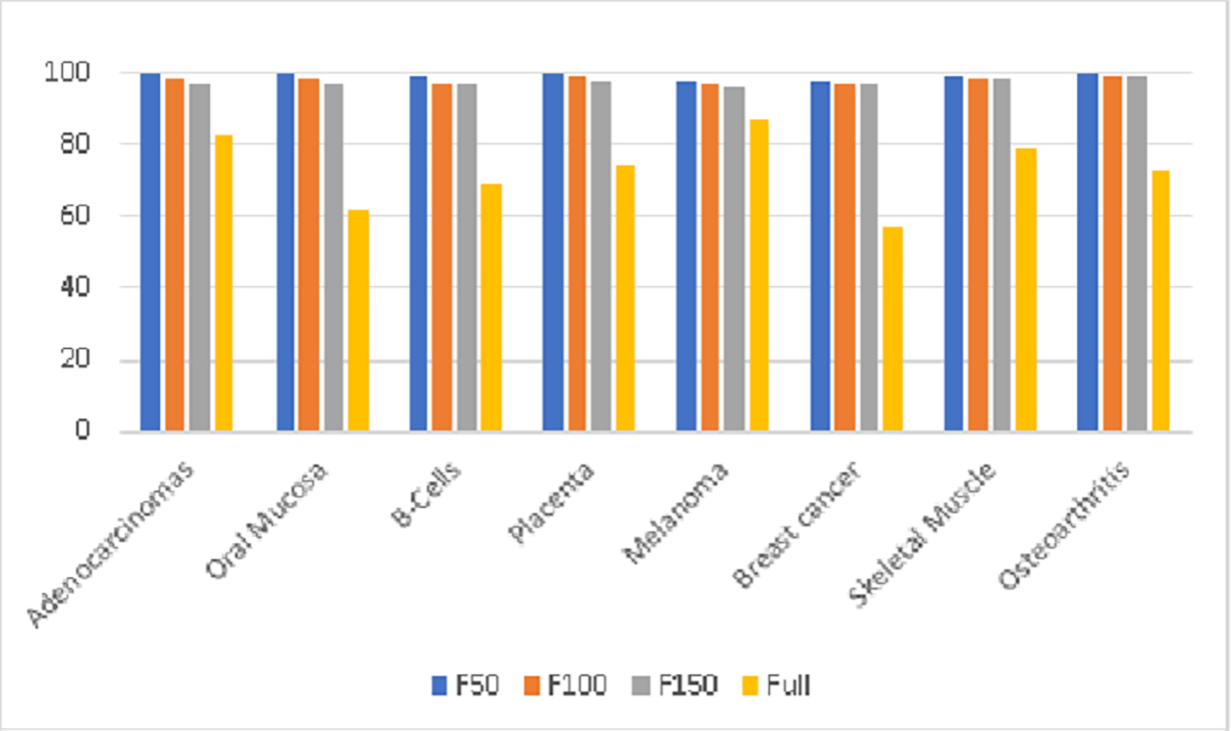
Comparison of GP performance on test dataset for 50 (F50), 100 (F100) and 150 (F150) features constructed by random projections and full feature set.

When we compare results from full feature set with constructed features, GP has shown significant an increase in overall accuracy with random projection-based constructed features as shown in Fig 2 and a decrease in standard deviation. For all the dataset there is an increase of 15% to 40%. For DT, there is a decrease of 5% to 15% in overall accuracy. For NB, there is a decrease of 2% to 5%. In case of KNNs, B-Cells, Melanoma, Adenocarcinoma and Osteoarthritis datasets have shown better results as that of the full feature set with an increase of 2% to 10%. For SVMs, there is an increase of 2% to 20% for most of the datasets except Adenocarcinoma, Oral mucosa, and B-cells. For RF, there is an increase of 1% to 7% in the overall accuracy of newly constructed feature sets for most of the datasets.

## Conclusion and future work

In the light of above results, it is evident that random projections are very effective for feature construction when combined with the genetic programming as a classifier. For future work, we will explore this method to address other high-dimensional problems like DNA-binding protein prediction [19], detection of tubule boundary [20], methylation site prediction [21], phosphorylation site prediction [22] and protein-protein interaction prediction [23,24], etc.
